# Construction and Validation of Institutional Identity Scale for University Students

**DOI:** 10.3390/children8080665

**Published:** 2021-07-30

**Authors:** Zunaira Bilal, Saba Ghayas, Taram Naeem, Sumaira Kayani, Ruibo Xie, Michele Biasutti

**Affiliations:** 1 Department of Psychology, University of Sargodha, Sargodha 40100, Pakistan; zunairabilal86@gmail.com (Z.B.); Saba.ghayas@uos.edu.pk (S.G.); taramch786@gmail.com (T.N.); 2 Department of Psychology, College of Education, Zhejiang Normal University, Jinhua 321004, China; xrb4526@zjnu.edu.cn; 3 Department of Philosophy, Sociology, Education and Applied Psychology (FISPPA), University of Padova, 35139 Padova, Italy

**Keywords:** institutional identity, commitment, crisis, validity, reliability

## Abstract

The current study presents the validation process of a measure of institutional identity for university students. The research is composed of two studies. Study I consisted of the generation of an item pool based on the literature review, semi-structured interviews, and expert opinion, which were administered to a convenient sample of university students (*n* = 707; 300 males and 407 females) in Pakistan. Exploratory factor analysis yielded a two-factor structure with 20 items, and the factors were named commitment (α = 0.84) and crisis (α = 0.74). The two-factor solution was confirmed through confirmatory factor analysis, which revealed an excellent model fit with the two-factor structure. Study II reports on the convergent and divergent validity of the scale which was carried out on an independent sample (*n* = 120). Results provided evidence of convergent validity as depression correlated negatively with the commitment subscale and positively with the crisis subscale. Divergent validity was ensured by a non-significant correlation between the subscales of the newly developed scale and a measure of religious belief. Moreover, the implications and limitations of the study are discussed.

## 1. Introduction

The drive to belong with others and feel being a member of a group, an institution, or a society is a natural process [1] which becomes particularly relevant among adolescents and early adults [2]. Previous research highlighted that a sense of connectedness or belonging has a relationship with many psychological-, emotional-, health-, and academic-related factors [3,4,5,6]. Conversely, the absence of a sense of belonging is a source of many adverse outcomes such as depression, suicidal ideation, loneliness, emotional distress, mental illness, and psychological disturbances [3].

In the contemporary era, a family is not the only social support against social factors. Besides family, school belonging is a prominent protective shield for children and adolescents against many psychological issues, such as eating disorders, emotional distress, and suicide [4,5]. Scholars suggest that school belonging is the most vital factor among many others that automatically decrease negative behavior patterns, such as absenteeism, inclination towards sexual activities, substance abuse, and violence in both girls and boys [5]. A positive correlation has been found in previous research between a sense of belonging and academic success and motivation level [7,8]. A sense of connectedness is also moderately correlated with good school attendance [9] and academic self-efficacy [10]. Similar patterns can be found among university students who have developed a solid institutional identity. A negative correlation has been found between anxiety and a sense of connectedness with the institution [8], induced by several situations.

The transition from high school to university is a challenging period. Challenges faced by students and their successful resolution led students to develop a sense of identification with their institute characterized as institutional identity. Institutional identity is a flexible phenomenon developed among members through their mutual institutional interactions to create a sense of belongingness to their institution that is considered different from other universities [11]. Researchers have identified the following factors that characterize institutional identity: (1) self-organization, (2) context, (3) seeing the self (or others), and (4) member’s sense of commitment. (1) Self-organization is an institute’s member desire to be perceived as a distinguished member of a particular group. This factor is responsible for strengthening one’s in-group ties [12]. (2) Context includes both human and contextual or social factors [13] and becomes a determining variable in changing a member’s attitudes and behaviors. (3) Seeing the self (or others) could be considered as an embodiment of the in-group prototype, which is named de-personalization, which makes every single member identify with the institution and thus support their institutional identity [14]. (4) Member’s sense of commitment is a crucial factor, which completes institutional identity [15] and plays a crucial role in fostering and strengthening the in-group ties and member’s loyalty towards institutions [16].

The literature provides evidence for additional aspects as core elements of institutional identities, such as the member’s personal choice of becoming involved in an institution [17] and the characteristics of the people in the institution [18]. The year of education, selection of roommates [19], perceived fairness, and relationship with the teachers [20] are additional factors in determining belongingness with institutes among students. Based on the literature, we can consider these crucial elements for developing an institutional identity among university students.

### 1.1. Marcia’s Model of Identity Status

Different theories have been proposed to describe identity formation. Upon reaching adolescence, an individual starts exploring to meet some standards that might help him in identity formation, which involves developing beliefs, values, attitudes, opinions, and capabilities. Erikson [21] proposes a theory based on the following eight psychosocial tensions that individuals have to resolve during their lives: trust vs. mistrust, autonomy vs. shame and doubt, initiative vs. guilt, industry vs. inferiority, identity vs. role confusion, intimacy vs. isolation, generativity vs. stagnation, and integrity vs. despair.

Considering Erikson’s concept of identity, Marcia [22] developed a new model termed the identity status paradigm. According to this model, an individual’s capabilities, ideas, opinions, beliefs, and history in different fields of life, such as religion, career, and sex roles, are depicted in terms of an individual’s inner self-structure. Crisis and choices are the core variables in Marcia’s identity status model, and it further leads towards differentiation among adolescents based on four identity statuses. Two dimensions play a crucial role in determining this structure: (a) crisis and exploration and (b) commitment. According to Marcia [22], crisis is a state of exploration and reassessment of values and choices by an individual. Commitment is considered a solution and an end to this crisis and could happen when an individual finds an answer to these questions of values, beliefs, and career options and committing to them. An individual deals with identity development through four strategic stages identified by Marcia [22] as identity achievement, foreclosure, moratorium, and diffusion. These stages are marked by the level of exploration, involvement, and commitment that an adolescent attempts to form identities in different domains, such as religion, education, gender roles, relationships, sex roles, vocation, and values [23].

An individual at the stage of identity achievement overcomes the crisis and makes a firm commitment, whereas those with diffused identity neither face any crisis nor are willing to commit [23]. We can define institutional identity as “an individual’s state of commitment and crisis towards an institute while being part of it.” It refers to how much an individual feels invested and connected with the institute and how much one is still confused and is in the state of exploration and making choices. Keeping in mind the importance of identity status regarding institutions, the current study was designed to develop a tool to measure the institutional identity of university students.

### 1.2. Measurement of Institutional Identity

School belonging, connectedness, and related concepts have been assessed using different measures. Ref. [24] identified more than 21 different tools that measure school belonging under different related terms, and we can find both qualitative and quantitative approaches. Regarding qualitative approaches, an open-ended questionnaire format was used to measure participants’ identity [25], including a few personal questions related to institutional identity, such as “Why are you teaching in ‘this’ high school? Do you have any feeling of belonging it’? Please explain”.

Regarding quantitative approaches, developed the 18-item Psychological Sense of School Membership Scale (PSSM), a famous and widely used tool by researchers and practitioners to measure the sense of connectedness and attachment to schools for middle and high school students [7]. This scale was developed to examine how students perceive their belongingness, and it has been found to be related to academic motivation in school. PSSM has been used by researchers, including a positive correlation between a sense of belonging and success and motivation at school [7,8]. Likewise, a study using The PSSM found a negative correlation between the PSSM score and the Child Depression Index [26]. Regarding the factorial structure, initially, it was a uni-dimensional scale. At the same time, other researches highlighted two dimensions [27] and three dimensions [28,29]. There has been extensive work on PSSM to define a perfect number of factors, and its internal consistency ranges between 0.77 and 0.88 across samples.

Institutional identity has also been measured among African American males in college in relation to self-esteem using an institutional identity self-report scale [30]. It is a 5-dimensional scale, each dimension having three items. Three of its dimensions, namely, centrality, private regard, and public regard, are the adaptations of the dimensions with the same name used in the Multi-dimensional Inventory of Black Identity [31]. Belonging and bonding are the dimensions that the authors created and added on their own for their study. The subscale positive regard (α = 0.738) assesses the positive feelings of a student towards his institute, the subscale public regard (α = 0.688) assesses one’s perception about other’s attitude towards his institute, the subscale centrality (α = 0.569) examines how central being a part of the institution is to one’s identity, the subscale belonging (α = 0.579) measures how much one feels to be a part of his academic institute, and, lastly, the subscale bonding (α = 0.573) assesses how much one feels connected with others in the same institute [30].

Despite the availability of several studies and tools that measure belonging, connectedness, attachment, and institutional identity, this construct has to be explored at the university level. No specific scale exists to date to measure institutional identity among university students. We can assume that the same antecedents and outcomes might be present behind developing institutional identity at the university level. Still, they have to be studied thoroughly in this particular context. Being young, university students are the central pillars of a nation that will lead to the country’s progress. The student university population must not go neglected, and a need for such an instrument has to be considered. There is a call to develop a scale that measures institutional identity in the Pakistani context and native language because things operate differently across cultures and contexts. Patterns of living, thinking, and attachment are not the same for any individual, especially when studying in two different cultures. Hofstede’s cultural dimension theory [32] supports the notion that no two cultures or their members are the same. According to this theory, society’s culture, being a unique entity, exerts a significant influence in determining people’s lives, the values they hold in their lives, and their values influence their behaviors.

### 1.3. Potential Correlates of Institutional Identity

Depression and religious beliefs are considered potential correlations in determining the construct validity of the Institutional Identity Scale. Identity formation is one of the eight stages of psychosocial development suggested by Erikson [21]. If successfully passed through, it brings a strong sense of self-confidence, academic achievement, and much more. If left in crisis, it may put individuals into a state of confusion, self-doubt, academic failure, and more importantly, take them into depression. Many adverse outcomes, such as depression, suicidal ideation, loneliness, emotional distress, mental illness, and psychological disturbances, root from the absence of a sense of belonging [3]. Moreover, institutions have now adopted a secular and liberal approach providing each student an equal and fair environment and opportunities.

#### Current Study

Although previous studies have focused on students’ belongingness to their institutes, none of them were focused on university students and how their institutional identity develops. University life is considered a hallmark of one’s academic career. There is a lot of hard work and planning to reach this stage, particularly for Pakistani students, since their life will be shaped by the university they graduated from. The current study aims to develop a scale of institutional identity for university students and assess the scale’s psychometric properties and is composed of two studies as follows.

Study I: Construction of the Institutional Identity Scale for University Students

Study I consisted of the construction of an identity scale for university students. It consists of three phases: generation of an item pool based on literature and interviews, exploratory factor analysis (EFA), and confirmatory factor analysis (CFA) of the scale.

Study II: Convergent and discriminant validation of the Institutional Identity Scale for University Students

Study II was carried out for the convergent and discriminant validation of the Institutional Identity Scale for University Students.

## 2. Materials and Methods

### 2.1. Participants and Procedure

The convenient sampling technique enabled us to collect 707 university students from different public and private universities in Pakistan. Participants consisted of *n* = 300 men and *n* = 407 women and their ages ranged between 19 and 29 years (*M* = 23.11, *SD* = 2.78). Participants in study II consisted of 200 students (*n* = 100 men and *n* = 100 women) and their ages ranged from 20 to 26 years (*M* = 22.65, *SD* = 6.16).

After obtaining formal permission from the departmental heads, students were personally contacted. They were briefed about the purpose of the research, and they were ensured that confidentiality of their information would be maintained. Students were interested in filling out the questionnaires, and there was a one hundred percent rate of return. Finally, participants were thanked for their cooperation.

### 2.2. Instruments

The following tools have been used in the current research: the Institutional Identity Scale for University Students (IISUS), the Depression subscale of DASS-21, and the Belief subscale of Short Muslim Practice and Belief Scale.

Institutional Identity Scale for University Students (IISUS). This is a 20-item scale developed for the Pakistani context, as reported below.

Depression subscale of DASS-21. The Urdu version of the depression subscale from the DASS 21-item version was used to assess depressive symptoms, originally developed by Lovibond and Lovibond [33] and translated by Farooqi and Habib [34]. This subscale is comprised of seven items that reveal the depressant states of an individual. Its four-point response format ranges between 0 = never and 3 = always. The internal consistency of subscales for the original DASS ranges between 0.91 and 0.97, whereas the translated version ranges between 0.94 and 0.97. The minimum possible score on the depression subscale was 0, while the maximum possible score was 21. The sum of scores obtained on all items was taken as an index of depression.

#### Belief Subscale of Short Muslim Practice and Belief Scale

The Urdu-translated version of the belief scale from the Short Muslim Practice and Belief Scale [35] was used to assess Islam’s religious beliefs among university students. The scale was initially developed by AlMarri, Oei, and Al-Adawi [36] and was translated and validated by Ghayas and Batool [35]. The subscale used in this study comprised five items on a 5-point Likert scale ranging from 1 = strongly disagree to 5 = strongly agree. The internal consistency of the whole original scale was 0.83. In contrast, in the translated version, the internal consistency of the full scale is 0.78; for the subscale practice, 0.80, and the subscale belief, it is 0.70. The minimum possible score on the religious belief subscale was one, while the maximum possible score was 25. The sum of scores obtained on all items was taken as an index of religious belief.

### 2.3. Data Analysis Techniques

SPSS Version 20 was used to analyze the data of the study. The scale’s factor structure, internal consistency, item correlation, and descriptive statistics were calculated. We have calculated Cronbach’s alpha for the reliability of tools. AMOS software was also used to run the confirmatory factor analysis that assured us the best fit factor structure of the data. Kaiser–Meyer–Olkin (KMO) and Bartlett tests were carried out to ensure the sample sufficiency of the data. The skewness and kurtosis of the data were checked to establish the normality of the data. Different indexes of verification were also run on the tested model. These indexes include Chi-square degrees of freedom, CFI, GFI, TLI, and RMSEA.

### 2.4. Procedure

Study I: Construction of the Institutional Identity Scale for University Students. The study consists of III phases.

Phase I: Generation of item pool. Empirical and deductive approaches were applied to generate an initial pool of items. To obtain an in-depth knowledge of our construct and its concept clarity, possibly relevant literature (e.g., Erikson [21]; Marcia [23]) was reviewed. Already-existing scales, relevant theories, and underlying concepts have been explored. Further, to gain information about institutional identity, university students were interviewed. The steps followed for the item pool generation were as follows:

1. Literature on institutional identity, organizational identity, and school belongingness was explored and consulted for item pool generation on Institutional Identity among University Students.
2. Students (*n* = 30) from different departments and different institutional backgrounds were interviewed to obtain an original view of the targeted population. Semi-structured interviews provided in-depth information about the institutional identity of university students.
3. An initial pool comprised 48 items that originated from literature, interviews, and previously existing scales. For expert opinion regarding the suitability and appropriateness of items with relevance to construct, sample, and cultural context, the items were presented to a committee, having expertise in scale construction, 38 items that fulfilled the criteria of construct validity, concept clarity, and understandability were retained with mutual consensus.
4. The Likert-type five-point response format was used for the Institutional Identity scale that ranged from 1 = strongly disagree to 5 = strongly agree. The Likert-type response format was chosen for its balanced nature against both poles and its freedom for the respondents to choose from a range of responses that best suit them.

Phase II: Exploratory Factor Analysis. Participants from different universities were approached using a convenient sampling technique. They were briefed about the purpose of the study, and it was assured to them that their information would be kept confidential.

Phase III: Confirmatory Factor Analysis. Questionnaires were given to the participants with informed consent. To ensure accuracy, participants were briefed that information will be used only for research purposes.

Study II: Convergent and Discriminant Validation of the Institutional Identity Scale for University Students. Study II aimed to validate the Institutional Identity Scale for University Students, and the convergent and divergent validity of the scale was carried out on an independent sample (*n* = 120).

## 3. Results

### 3.1. Results of Study I

To ensure the construct validity of the newly developed scale, exploratory factor analysis was carried out on the 38 items using SPSS Version 20 [37]. Principal axis factoring with the direct oblimin rotation method was chosen for the EFA of the data of 707 students because of the expected significant negative correlation between the latent constructs [38], i.e., in the presence of high levels of commitment, the level of crisis will ultimately decline, and vice versa. The satisfactory level of the Kaiser–Meyer–Olkin (KMO) test and Bartlett tests provided evidence for the suitability of data for factor analysis. The KMO value = 0.89 revealed the excellent sampling efficiency for further analysis [39]. Bartlett’s test of sphericity (*χ^2^* = 3777.0319, *df* = 190, *p* = 0.000) was significant, revealing a significant correlation matrix, i.e., items are significantly correlated to each other and data are ready to be processed through further factor analysis [38]. The skewness and kurtosis values ensured the normal distribution of data. The communalities also became considerably higher, suggesting all the variables suitable for further factor analysis [40].

Initial exploratory factor analysis revealed a two-factor solution with a direct oblimin rotation method with eigenvalues >1.0. The derived factors were clear, interpretable, well defined with definite boundaries, and had theoretical reliability and relevance with the construct. A two-factor structure was retained with 38.71% of variance explained (Table 1). The threshold for factor loading was 0.40 [41,42]. Out of the initial 38 items, 20 items were retained based upon their substantial and satisfactory loadings (≥0.40) on every single factor. The remaining items were excluded either because of their low and unsatisfactory loadings or cross-loading on more than one factor. A two-factor solution was finalized for interpretation considering the variance, scree plot, factor loading and theoretical relevance of each item to the respective factor.

Twelve items showed exclusively independent loading in Factor I. “Liking,” “attachment,” “bondedness,” and “satisfaction” towards the institute were considered as hallmarks of this factor and hence, was named as “commitment.” Eight items were exclusively loaded in Factor II. “Detachment,” “comparison,” and “decline in self-confidence” in relevance to the institute constituted this factor and, hence, was named as “crisis.” Factor structure was finalized based on empirical and theoretical grounds.

Table 2 shows that both the subscales of commitment and crisis in the Institutional Identity Scale are reliable, having Cronbach alpha values of 0.84 and 0.74, respectively. The table shows that both the subscales are significantly negatively correlated (*r* = −0.39, *p* < 0.01).

**Figure 1:**
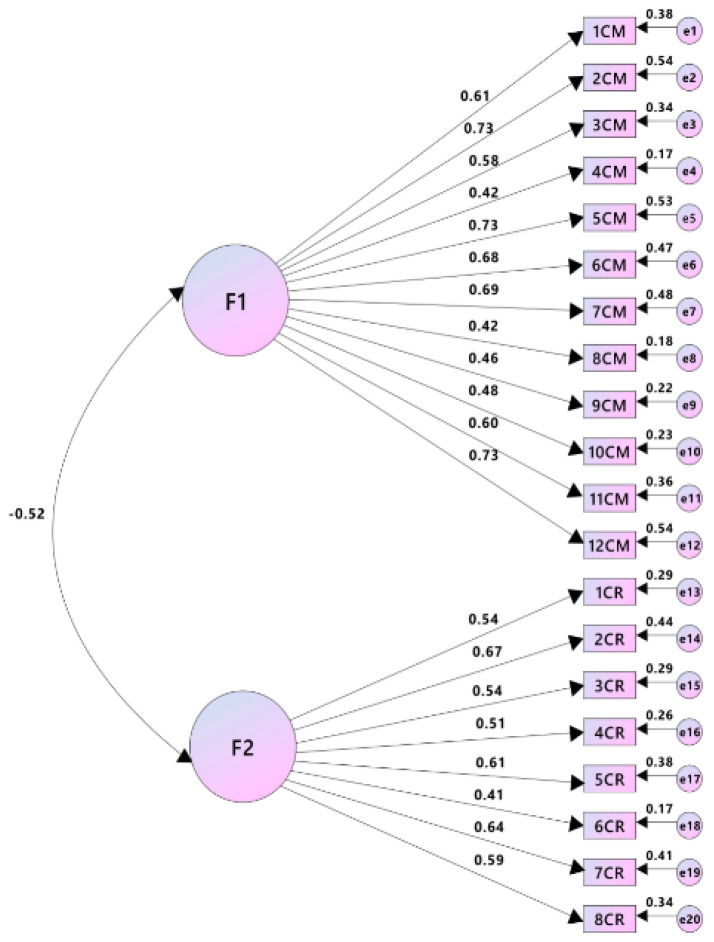
Measurement model of the Institutional Identity Scale for University Students.

Factors obtained from EFA were verified with CFA on a sample used in the third phase of the study to confirm the measurement model of the scale [43] using AMOS Version 20.0 (Figure 1). Keeping the item loading criteria the same as the initial one, i.e., >0.40, the two-factor solution was examined through CFA (Table 3), which showed that the yielded two-factor structure was a good fit to the data with a Chi-square value of 413.2 (*df* = 169) < 0.01, CFI = 0.90, GFI = 0.90, TLI = 0.90, and RMSEA = 0.04. A Chi-square value is required to be non-significant for CFA; however, it is also almost impossible for it to be non-significant in the case of a large sample size. Therefore, it is suggested to divide the Chi-square value by its degree of freedom to check if the model shows a good fit in the case of a significant Chi-square value, which is definite to occur in a large sample size. The Chi-square/*df* when it is below three is considered good [44], and in the current study, it was 2.4, which shows a good fit for the model as it lies within the recommended acceptable range.

The final model obtained through CFA was a good fit model and had 20 items in it; 12 for commitment with acceptable factor loadings (0.61, 0.73, 0.58, 0.42, 0.73, 0.68, 0.69, 0.42 0.46, 0.48, 0.60, and 0.73, respectively), and 8 items for the crisis which also appeared to have acceptably high factor loadings (0.54, 0.66, 0.54, 0.51, 0.61,.41 0.64, and 0.59, respectively). The factor loadings of all the items ranged between 0.41 and 0.73.

### 3.2. Results of Study II

Table 4 reveals the construct validity of the scale in terms of convergent and divergent validities. The table shows that depression has a significant positive correlation with crisis and a significant negative correlation with commitment. Religious belief was found to have a non-significant correlation with the institutional identity subscales. Results provided strong evidence for the construct validity of the Institutional Identity Scale.

## 4. Discussion

Identity formation has been considered as one of the vital parts of an individual’s life. According to Erikson [21], identity formation is not confined to teenage years or adolescence. Conversely, it develops throughout life whenever a person is confronted with challenges and has to deal with different life experiences. Marcia expanded Erikson’s concept of identity in terms of the Identity Status Paradigm [22] as commitment and crisis. In the current study, this identity formation was studied in the university student context under the title of Institutional Identity. Keeping Marcia’s identity status paradigm in mind, institutional identity is a state of commitment and crisis towards an institute while being in it [45], that is, how much an individual feels invested and connected with the institute and how much one is in the state of exploration and making choices. This phenomenon has been studied in the contexts of school and organization but was lacking for university students.

The current study is an advancement in this direction as it aims to develop a psychometrically sound instrument in terms of reliability, validity, and factorial structure. Exploratory factor analysis with the oblique rotation method was carried out on 38 items. Results yielded a two-factor structure with an eigenvalue >1 and the inflection of scree plot at two factors. Following the Kaiser criterion of factor extraction, two factors were retained with 38.71% of variance explained and were considered theoretically consistent with Marcia’s Identity Status Paradigm [22]. Factors were named “commitment” and “crisis.” The Institutional Identity Scale obtained acceptable and high factor loadings ≥0.40, communalities >0.30, and high internal consistency for the two factors. The relevant factor solution with the pertinent model, confirmation through construct validity, and confirmation of factor structure on an independent sample through CFA showed a good model fit. The indices of CFA revealed that all the indicators have loadings on their respective factors. The two-factor structure obtained with the EFA was confirmed through CFA, showing a good model fit for 20 items.

The commitment factor (12 items) yielded as a result of EFA. It was replicated when put in CFA and sounded quite reliable and valid, and it measures the extent to which an individual expresses his investment in his occupation or belief [45]. It is about how deeply and keenly an individual exhibits his investment into his personal and occupational ideology. A person who has reached a commitment level has made strong convictions and choices [46]. This commitment stage has either been reached directly without any exploration or has been directed to facing and resolving the crisis. According to Marcia [22], individuals with commitment towards any aim or choice have been through a stage of exploration or crisis. Once the commitment is made, the individuals are all clear about the institution they have enrolled into, the courses they opted for, and all other choices they made related to their institute and are all willing to put all their efforts into this pursuit. No doubt is left to consider alternatives or explore more options. They trust their choice, become close to the people around them, and keep excelling in their field with confidence.

The crisis factor (eight items) yielded as a result of EFA and then replicated when put in CFA and became quite reliable and valid. It measures how individuals are actively involved in exploring and creating crises among multiple, available, and alternative occupations and beliefs [45]. It is a state wherein individuals are actively trying out all the possible options and have not yet made a final choice. They have not yet developed a sense of belonging and attachment with the institute. The individuals in a crisis state are still searching for values that they can own or call their own. According to Marcia, the crisis is a state of continuous struggle searching for an identity that might lead to commitment. The individual struggling in crisis is weighing the available identity alternatives [46]. Individuals in crisis are not satisfied with their choice but are still considering the other available options for universities, programs, and courses they should be enrolled into. They are confused about whether the field or university they chose is suitable, best, or helpful in achieving their future goals, and hence, are unable to own the institute they are currently enrolled in. They develop belonging and attachment with the people around them and keep lacking their inner self-confidence.

The current results revealed that the Institutional Identity Scale for University Students had sufficient internal consistency and was quite reliable and valid. The two factors of the scale were significantly negatively correlated with each other. The negative relationship among the subscales was in line with the previous literature. According to Marcia [22], there is an exploration and reassessing of values and choices by an individual in a state of crisis, and there is no commitment. When the individual commits, it brings an end to that crisis state by answering values, beliefs, and career options and committing to them. In a state of commitment, individuals have to overcome the crisis. Thus, the stable, interpretable, and satisfactory indices of the two-factor solution show that the Institutional Identity Scale is a reliable and valid measurement tool.

The Institutional Identity Scale’s construct validity has been supplemented by the findings wherein commitment had a significant negative correlation and crisis had a significant positive correlation with depression. Commitment is negatively correlated with depression because commitment is a state that an individual has achieved by overcoming hardships, crises, and confusion. This is a state of finally doing something in pursuing final choices. This is the state when an individual has made a resolution and found their answers to the questions of values, beliefs, and career options and commit to them. In the case of commitment to the university or institute, department, and field of study, individuals confidently determine their plans and invest all their efforts in this pursuit. They become satisfied with the choices they made and start making an effort to get closer to the people around them. They appreciate their choices and are willing to take a stand for them. Such commitment and sense of belonging to the institute leads to many psychological-, emotional-, health-, and academic-related positive outcomes while ruling out the chances of depression [3,4,5,6]. A higher score on the Psychological Sense of School Membership Scale yields a higher score in school success [7,8] and a lower score in depression [26].

Crisis has a positive correlation with depressions, and according to Marcia [22], crisis is a state of turmoil wherein individuals are exploring and reassessing their choices and values. The individuals have not yet determined their choices regarding the program they want or should attend. The decisions they are making are only trial and error based. For example, the students studying psychology might not yet be sure about if they want to pursue their career as a psychologist or not. They are still exploring and trying to see if this field fits their area of interest. Continuing to realize that this field does not fit their interest leads to frustration and detachment and eventually leaves them in a state of depression. The absence of a sense of belonging is a source of many adverse outcomes, such as depression, suicidal ideation, loneliness, emotional distress, mental illness, and psychological disturbances [3]. McAdams and Bryant [47] have seen a strong positive relationship between an individual’s belonging with others around him and increased happiness and psychological well-being. Likewise, lack of belonging, social bonding, and an explicit feeling of being left out contribute to increased feelings of anxiety [48] and other related psychological outcomes, such as depression, social anxiety and loneliness, that become reduced upon the development of a sense of belonging among college students [49]. In addition, it was found that school belongingness is a robust negative predictor of depression among adolescents [50]. Recently, researchers also support the notion that higher levels of a sense of belonging in college students lead to decreased mental health problems [51].

In the scenario of discriminant validity, the second hypothesis narrates that religious belief will be non-significantly correlated with both commitment and crisis. This hypothesis was supported as commitment and crisis did not correlate with religious belief. This result provided empirical support to the discriminant validity of the Institutional Identity Scale. The same pattern has been considered in other studies [52], highlighting that religious identity, belief, or commitment did not correlate with school belonging, teacher–student relation, and valuing education. Commitment and crisis can be stated as independent of one’s religious beliefs.

## 5. Conclusions

The Institutional Identity Scale developed in this study has shown to be a reliable and valid tool. The scale and its subscales demonstrated a satisfactory level of reliability and internal consistency. This scale indicates the factors that determine a university student’s institutional identity. The Institutional Identity Scale determines the domains to be considered when identifying and measuring a student’s institutional identity.

## 6. Limitations and Further Suggestions

This study holds certain limitations that must be addressed in future research. This study did not maintain an equal proportion of students from different universities, influencing the results, and this issue could be considered when designing further research. In addition, data were collected only from one province of the country, and findings could not be generalized to all university students. Hence, diverse and equally proportioned data needs to be collected in future studies. Therefore, it is suggested to translate and validate the scale in other languages so that it may be used to measure students’ institutional identity in other countries.

## 7. Implications of the Study

The development of the Institutional Identity Scale as an empirically and psychometrically sound scale might lead to new research avenues. In future research, this scale can be used for measuring and identifying institutional identity among university students. This scale can help to identify the reason for depression and distress students experience during their university careers. This scale could be useful in identifying the expected success outcomes among university students, helping them in overcoming the crisis. Academic professionals could be aided with this scale in identifying the state of students, whether it is a commitment or a crisis state.

This scale could help study counselors identify students who are experiencing isolation and feelings of rejection within their university community. It helps identify the possible factors behind students’ continuous absenteeism, failure, or disputes. Once targeted, such students can be helped by additional support by giving them career counseling, expanding their social circles, and increasing feelings of acceptance. This study will guide the tricks to reduce the feelings of depression among students by helping them to find a resolution to their conflicts. The newly developed scale could guide university authorities to create such an environment and train the teachers to provide the facilities to students that are conducive for developing institutional identity by making it easy for them to commit and overcome the crisis and conflicts.

## Figures and Tables

**Table 1:** Factor loading of 20 items of Institutional Identity Scale for University Students for two factors in factor solution obtained through Oblique rotation (*N* = 707).

New No. of Items	Factors	
	I Commitment	II Crisis
1	0.51	−0.40
2	0.62	−0.36
3	0.59	−0.31
4	0.52	−0.21
5	0.71	−0.32
6	0.67	−0.39
7	0.67	−0.29
8	0.50	−0.16
9	0.56	−0.01
10	0.53	−0.21
11	0.65	−0.15
12	0.76	−0.33
13	−0.33	0.57
14	−0.27	0.60
15	−0.20	0.58
16	−0.20	0.51
17	−0.13	0.59
18	0.10	0.50
19	−0.19	0.66
20	−0.22	0.63
Eigenvalues % of variance explained	5.56	2.18
	27.81	10.90

**Table 2:** Correlation matrix of subscales means, standard deviations, and alpha reliabilities of the Institutional identity Scale for University Students (*N* = 707).

Variables	Commitment	Crisis	*M*	*SD*	*α*
Commitment	--	−0.39 **	42.47	7.38	0.84
Crisis		--	22.64	5.54	0.74

** *p* < 0.01.

**Table 3:** Model fit indices of CFA for the Institutional Identity Scale for University Students (*N* = 200).

Indexes	Chi-Square	*df*	Chi-Square/*df*	CFI	GFI	RMSEA	TLI
Model	413.26	169	2.4	0.90	0.90	0.04	0.90

**Table 4:** Inter-correlations among study scales (*N* = 120).

Variables	Identity Commitment	Identity Crisis
Belief	0.06	0.06
Depression	−0.19 ***	0.41 ***

*** p < 0.001 or *p* > 0.05.

## Data Availability

The data presented in this study are available on request from the first author.
